# Vexatious litigant vs paranoia querulans: A systematic review

**DOI:** 10.1192/j.eurpsy.2021.1023

**Published:** 2021-08-13

**Authors:** J. Pinzón-Espinosa, A. González-Rodríguez, A. Guàrdia, M. Betriu Sabaté, P. Manozzo-Hernandez, A. Alvarez Pedrero, S. Acebillo, J. Labad, D. Palao Vidal

**Affiliations:** 1 Salut Mental Taulí, Parc Taulí Hospital Universitari, Sabadell, Spain; 2 Department Of Clinical Psychiatry, School of Medicine, University of Panama, Panama City, Panama; 3 Department Of Medicine, School of Medicine, University of Barcelona, Barcelona, Spain; 4 Mental Health, Parc Taulí University Hospital. Autonomous University of Barcelona (UAB). I3PT. CIBERSAM, Sabadell, Spain; 5 Mental Health, Parc Taulí-University Hospital, Sabadell, Spain; 6 Mental Health, Parc Tauli University Hospital, Sabadell, Spain; 7 Mental Health, Parc Taulí University Hospital. Autonomous University of Barcelona (UAB). I3PT, Sabadell, Spain; 8 Mental Health, Hospital of Mataró. Consorci Sanitari del Maresme. CIBERSAM., Mataró, Spain

**Keywords:** Paranoia querulans, Vexatious litigant, Psychiatry and Law, Delusional disorder

## Abstract

**Introduction:**

Paranoia querulans is a type of persistent delusional disorder of the persecutory subtype, recognized under ICD-10 and DSM-IV. Being a classically described entity, evidence is lacking from its conceptualization as a nosological entity to diagnosis and treatment. Furthermore, controversy still exists regarding its interplay between the judicial and mental health systems.

**Objectives:**

To summarize current evidence and knowledge regarding Paranoia querulans on its conceptualization, ethiopathological explanations, therapeutical management and interface between psychiatry and the law.

**Methods:**

A systematic review was undertaken between June and October 2020 in the PubMed, Web of Science and Scopus databases according to PRISMA directive. Key-terms: ((querul* OR vexatious) AND (paranoia OR delusio* OR neuros* OR behavi* OR complai*) OR litig*) AND psychiatry. No language or time restrictions were established.

**Results:**

A total of 1648 studies were initially identified (PubMed: 679; WOS: 945; Scopus: 24; other: 0); after duplicates were removed, n=1381 studies remained. After screening title and abstract, 56 studies were included. Their main content was categorized into: 1. Conceptualization (n=26): Neurosis (n=5), psychosis (n=9), behavioral disorder (n=5); no psychiatric diagnosis (n=7). 2. Descriptive psychopathology (n=8) 3. Etiopathogenesis (n=9): Social or personality basis (n=3), culture (n=4), trauma (n=1), cognitive decline (n=1) 4. Management (n=1) 5. Psychiatry and Law: same object, different objectives (n=12)

**Conclusions:**

There is controversy regarding the nosological entity of querulousness, from psychosis to neurosis or behavioral disorders. Some authors consider this behavior to not be a psychiatric diagnosis. Furthermore, most papers dealt with a social or nurture-based origin. There is a dearth of information regarding treatment.

**Conflict of interest:**

JPE has received CME-related fees from Lundbeck.

